# Study on Biological Characteristics and Mechanism of Paclitaxel Induced Drug Resistance in Endometrial Carcinoma Cells

**DOI:** 10.1155/2018/8372085

**Published:** 2018-08-05

**Authors:** Jie Ding, Mengxiong Li, Liuzhi Deng, Tian Li

**Affiliations:** ^1^The Third Affiliated Hospital, Sun Yat-sen University, Guangdong Province, China; ^2^The Seventh Affiliated Hospital, Sun Yat-sen University, Guangdong Province, China

## Abstract

**Objective:**

To study the biological characteristics of paclitaxel resistant endometrial carcinoma cells and its mechanism of drug resistance.

**Method:**

The paclitaxel resistant cell lines were established by high-dose paclitaxel (TAX) injection. The IC50 of paclitaxel was determined by CCK-8 assay in Ishikawa and Ishikawa-TAX. The cell cycle and apoptosis rate were detected by flow cytometry. Western blot was used to detect the expression of p-AKT and p-p70S6K. The expression of drug resistance-related genes Pgp and MDR1 was determined by RT-PCR. Cell viability was determined by soft agarose assay and invasive ability in vitro by transwell assay.

**Results:**

Paclitaxel and NVP-BEZ235 cotreatment group can further inhibit the clonogenicity and invasion of Ishikawa and Ishikawa-TAX cells compared with paclitaxel alone and NVP-BEZ235 treatment group. Paclitaxel and NVP-BEZ235 cotreated groups increased the apoptosis rate of Ishikawa and increased G0/G1 phase arrest in both cells. Paclitaxel alone significantly inhibited p-AK and p-p70 S6K protein expression in Ishikawa and Ishikawa-TAX cells and the inhibition was enhanced by NVP-BEZ235 when cotreated with paclitaxel.

**Conclusion:**

Paclitaxel can inhibit Ishikawa and Ishikawa-TAX cell via PI3K/Akt/mTOR signaling pathway. Paclitaxel and NVP-BEZ235 cotreatment can enhance the inhibitory effect.

## 1. Introduction

Endometrial cancer is one of the most common gynecologic malignancies. The mortality is increasing because of the lack of no effective treatment for advanced and recurrent endometrial cancer, which seriously affects the quality of life of women [1, 2]. At present, chemotherapy is the primary method of treatment [3]. The combination of paclitaxel/taxol (TAX) and platinum compounds is the first-line chemotherapy regime for endometrial cancer, which shows a certain effect on primary endometrial cancer [4]. Paclitaxel is an antimicrotubule agent, which binds to the h-tubulin subunit and stabilizes the microtubules, resulting in disruption of microtubule dynamics during cell division [5]. However, with the extension of dosage and duration, the induced paclitaxel resistance often leads to the failure of chemotherapy in endometrial carcinoma [6]. Therefore, the study of drug resistance in endometrial cancer is of great significance for clinical treatment, especially for the advanced and recurrent endometrial cancer.

PI3K/mTOR signaling pathway is frequently activated in a variety of human tumors, such as breast cancer, ovarian cancer, and bladder cancer [7–9]. In recent years, the study showed that the abnormal activation of phosphatidylinositol-3 kinase/protein kinase B/mammalian target of rapamycin (PI3K/Akt/mTOR) signaling pathway (e.g., gene mutation) can inhibit the apoptosis of endometrial cancer cells and promote cancer cell proliferation, invasion, and angiogenesis [10, 11]. Therefore, the biological characteristics of paclitaxel resistant endometrial cancer cells and their correlation with PI3K/mTOR signaling were studied in this paper, so as to provide a theoretical basis for clinical treatment of paclitaxel resistant endometrial cancer.

## 2. Materials and Methods

### 2.1. Cell Culture

The endometrial carcinoma cell line (Ishikawa) was purchased from Shanghai cell bank of Chinese Academy of Sciences. Ishikawa cells were cultured in RPMI1640 culture medium containing 10% FBS, 1% penicillin, and 100 U/ml streptomycin, at 37°C and 5% CO_2_.

### 2.2. Construction of Paclitaxel Resistant Cell Line (Ishikawa-TAX Cells)

The MTT method was used to detect IC50 in Ishikawa cells. When the parent cell confluence reached 80%, the cells are treated with taxol (sigma, USA) at the concentration of 1/10 IC50. The concentration of taxol was kept in the culture medium by changing medium with taxol. When cell growth was stable in the low concentration taxol medium (1/10 IC50), the taxol concentration was raised gradually until 5-10 times of IC50. The treated Ishikawa cells grew well with the high concentration of taxol and therefore the paclitaxel resistant cell line (Ishikawa-TAX cells) was successfully established.

### 2.3. NVP-BEZ235 and Paclitaxel Treatment

When cells grew to 80% density, NVP-BEZ235 (MedChemExpress, USA) was added to medium at concentration of 5 nM. After 6 hours, TAX was added to 0.01 ug/ml for 24 hours, then the cells were collected for follow-up experiments.

### 2.4. MTT Assay

Cells at logarithmic growth phase were collected and suspended at the concentration of 10^4^-10^5^ /ml. 100 ul of cell suspension was added to each pore of 96-well plate and MTT assay was carried out on the corresponding day. 20 ul MTT solution was added and the cells were incubated for 4 h. The supernatant was then carefully discharged and 150 ul DMSO was added to dissolve the crystallization. The absorbance at OD492 nm was measured by Microplate Reader.

### 2.5. Clone Formation

Cell suspension was prepared by trypsin-EDTA digestion and cell density was adjusted according to the number of Trypan blue positive staining cells. 100 living cells were inoculated in each hole of 6-well plates and then the cells were cultured for 15 days at normal condition. At day 15, the culture medium was discharged and cells were fixed by paraformaldehyde. After washing by PBS, the cells were stained by crystal violet for 10 min. The number of clones was counted. All experiments were conducted in triplicate.

### 2.6. Cell Apoptosis Detection

Cells were harvested by trypsin and collected by centrifugation at 400×g for 5 minutes. A kit from BD company was used for cell apoptosis analysis. Carefully decant all the supernatant, and add 250 *μ*L of Solution A (trypsin buffer) for 10 minutes and 200 *μ*L of Solution B (trypsin inhibitor and RNase buffer for) 10 minutes. Do not remove Solution A and Solution B. Add 200 *μ*L of cold Solution C (propidium iodide stain solution); incubate for 10 minutes in the dark in the refrigerator (2° to 8°C). Cell apoptosis was analyzed using flow cytometry and Cell Quest software. All experiments were conducted in triplicate.

### 2.7. Cell Cycle Detection

Cells were collected and washed with cold PBS twice, resuspended in 1×binding buffer at a concentration of 10^5^-10^6^ cells/ml, and transferred 100 *μ*l of cell suspension to a 5 ml flow cytometry tube. 5 *μ*l of Annexin V-PE was added to the cell suspension and incubated for 15 min in dark at room temperature. Another 400 *μ*l of binding buffer was added and then analyzed by flow cytometry. All experiments were conducted in triplicate.

### 2.8. Transwell Assay

Matrigel was dissolved at 4 C overnight and diluted with precooled serum-free medium at a volume ratio of 1:3. 40 ul Matrigel was added to a precooled transwell chamber and incubated at 37 C for 2 h to solidify. The excess liquid in the chamber was discharged, counted the cells carefully, and resuspended 1*∗*10^5^ cells in 200 ul serum-free media. Cell suspension was added to the upper chamber and 1000 ul complete medium was added to the lower chamber. After incubation for 24 hours at 37°C and 5% CO_2_, the chamber was removed and the cells of the upper chamber were wiped away with a cotton swab. The cells in lower chamber were fixed by 4% polyformaldehyde 15 min and then washed by PBS and stained by crystal violet. All experiments were conducted in triplicate.

### 2.9. Western Blot

Cells were harvested, lysed, and centrifuged at 15,000 rpm for 5 min at 4°C. Protein content in the supernatants was determined by a BCA protein assay kit [12]. Equal amounts of protein were separated by 10% SDS-PAGE, transferred onto PVDF membranes, and then incubated with p-AKT (1:500, Santa Cruz, USA), p-p70S6K (1:500, Santa Cruz, USA), and *β*-actin (1:2000, Santa Cruz, USA) antibody overnight at 4°C. After incubation with secondary antibody (KPL, Gaithersburg, MD), membrane was treated with ECL-Western blot detecting reagent (Amersham Biosciences KK, Tokyo, Japan). Protein bands detected were estimated using Quantity One software (Bio-Rad Laboratories, Hercules, CA). The density measurement was correlated with protein expression and normalized to *β*-actin. All experiments were conducted in triplicate.

### 2.10. Quantitative Real-Time PCR

Total mRNA was extracted with TRIzol reagent (Invitrogen). cDNA was then generated from 1.0 *μ*g of total RNA with avian myeloblastosis virus reverse transcriptase (Takara, Otsu, Japan). Amplification was carried out with an ABI PRISM 7000 (Applied Biosystems, Tokyo, Japan) using SYBR green reagent for detection according to the protocol of SYBR Premix Ex Taq™ kit (Takara) [13]. All experiments were conducted in triplicate.

Primer sequences for Pgp were 5′GGAGAGATCCTCACCAAGCG-3′(sense) and 5′-CGAGCCTGGTAGTCAATGCT-3′(antisense). Primer sequences for MDR1 were 5′-TTTGGAGCCTACTTGGTGGC-3′(sense) and 5′-GCTTTGGCATAGTCAGGAGC-3′(antisense). Primer sequences for Caspase-3 were 5′-TGCATACTCCACAGCACCTG-3′(sense) and 5′-TCAAGCTTGTCGGCATACTGT-3′(antisense). Primer sequences for MMP-9 were 5′-GTACTCGACCTGTACCAGCG-3′(sense) and 5′-AGAAGCCCCACTTCTTGTCG-3′(antisense). Primer sequences for *β*-actin were 5′-GCATGGGTCAGAAGGATTCCT-3′(sense) and 5′-TCGTCCCAGTTGGTGACGAT-3′(antisense).

### 2.11. Statistical Analysis

All the experiments were repeated 3 times independently. Data were expressed as x + s and were analyzed by SPSS 17 software. Multiple groups were compared by ANOVA, and post hoc comparisons were carried out by LSD method. The difference was statistically significant when* P* < 0.05.

## 3. Results

### 3.1. Identification of Ishikawa-TAX Resistant Cell Lines and Their Proliferation Rate

**Figure 1:**
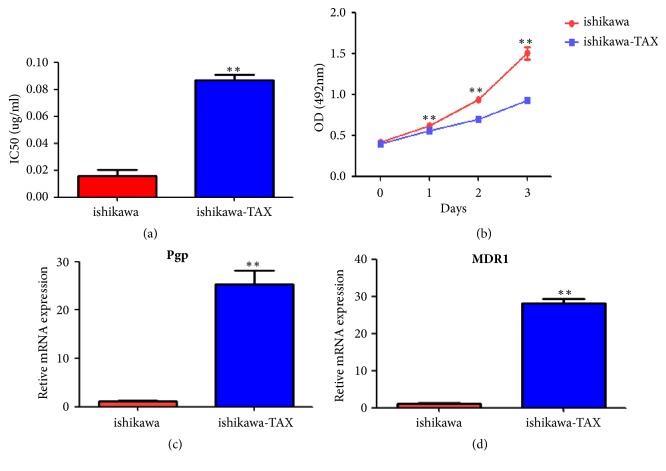
Identification of Ishikawa-TAX resistant cell lines and their proliferation rate. (a) IC50 test of Ishikawa and Ishikawa-TAX cells. (b) Cell proliferation assay of Ishikawa and Ishikawa-TAX cells. (c) Ishikawa and Ishikawa-TAX resistance-related gene test (P <0.05).

The results of CCK-8 showed that IC50 of Ishikawa-TAX group was significantly higher than that of Ishikawa group (Figure 1(a)). The proliferation rate of Ishikawa-TAX group was lower than Ishikawa group (Figure 1(b)). QPCR results showed that the resistance-related genes of Pgp and MDR1 were significantly higher in Ishikawa-TAX group than in Ishikawa group (Figure 1(c)).

### 3.2. NVP-BEZ235 Cotreatment with Paclitaxel Can Further Enhance Paclitaxel Inhibition of Ishikawa and Ishikawa-TAX Cloning Formation

**Figure 2:**
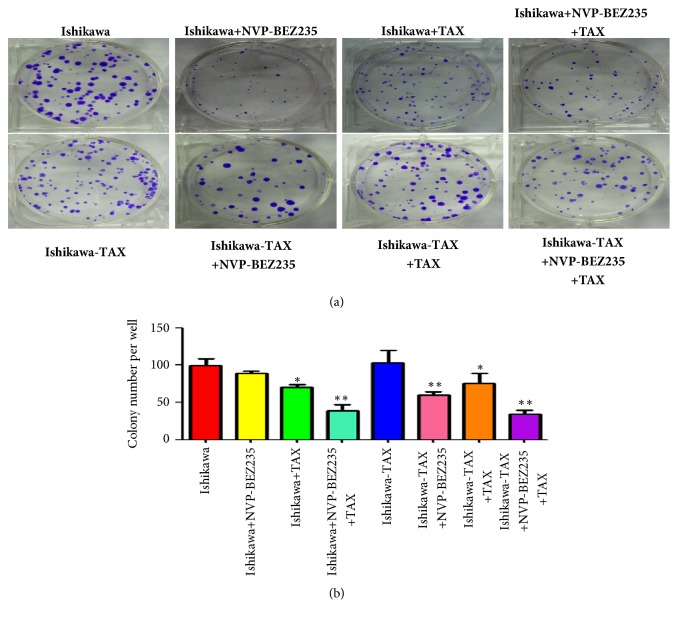
Clonogenicity after treatment of Ishikawa and Ishikawa-TAX cells with NVP-BEZ235, and paclitaxel. (a) Cell cloning following treatment of Ishikawa and Ishikawa-TAX cells with NVP-BEZ235 alone and in combination with paclitaxel. (b) Clone formation quantification (*∗*P <0.05; *∗∗*P <0.01).

Clone formation results showed that treatment with NVP-BEZ235 alone did not inhibit Ishikawa clonal formation, whereas paclitaxel alone inhibited Ishikawa clonality, and cotreatment with paclitaxel NVP-BEZ235 further inhibited Ishikawa clonality (Figure 2). However, in Ishikawa-TAX, NVP-BEZ235 treatment and paclitaxel treatment inhibited Ishikawa clonogenicity, whereas NVP-BEZ235 and paclitaxel further inhibited clonogenicity of Ishikawa-TAX (Figure 2)

### 3.3. NVP-BEZ235 Cotreatment with Paclitaxel Can Further Inhibit the Invasion of Ishikawa and Ishikawa-TAX Cells

**Figure 3:**
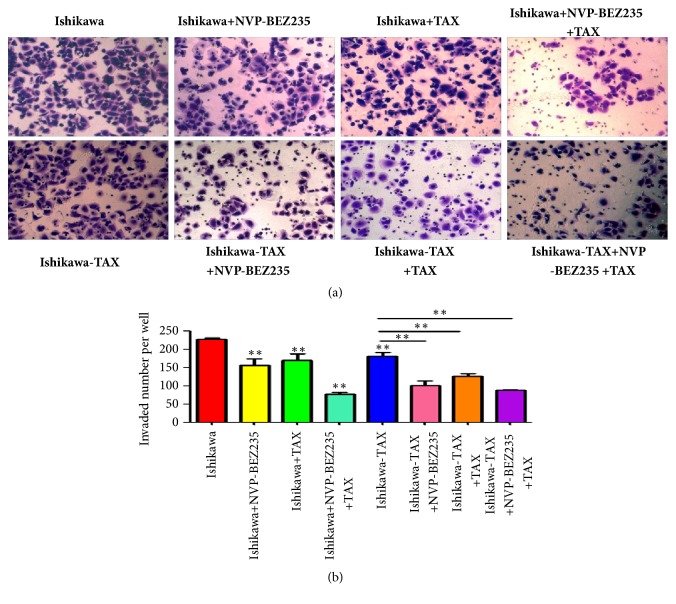
The cell invasion of Ishikawa and Ishikawa-TAX cells after NVP-BEZ235 treatment with paclitaxel. (a) The cell invasion of Ishikawa and Ishikawa-TAX cells treated by NVP-BEZ235 alone and cotreated with paclitaxel treatment. (b) The number of cell invasion was quantified (*∗∗*P <0.01).

Invasion results showed that NVP-BEZ235 alone and paclitaxel alone inhibited Ishikawa invasion, and NVP-BEZ235 cotreatment with paclitaxel further inhibited Ishikawa invasion (Figure 3). In Ishikawa-TAX, NVP-BEZ235 alone and paclitaxel alone inhibited the invasion of Ishikawa, whereas NVP-BEZ235 cotreated with paclitaxel further inhibited Ishikawa-TAX invasion (Figure 3)

### 3.4. NVP-BEZ235 Cotreatment with Paclitaxel Can Significantly Lead to an Increase in Apoptosis Rate Ishikawa

**Figure 4:**
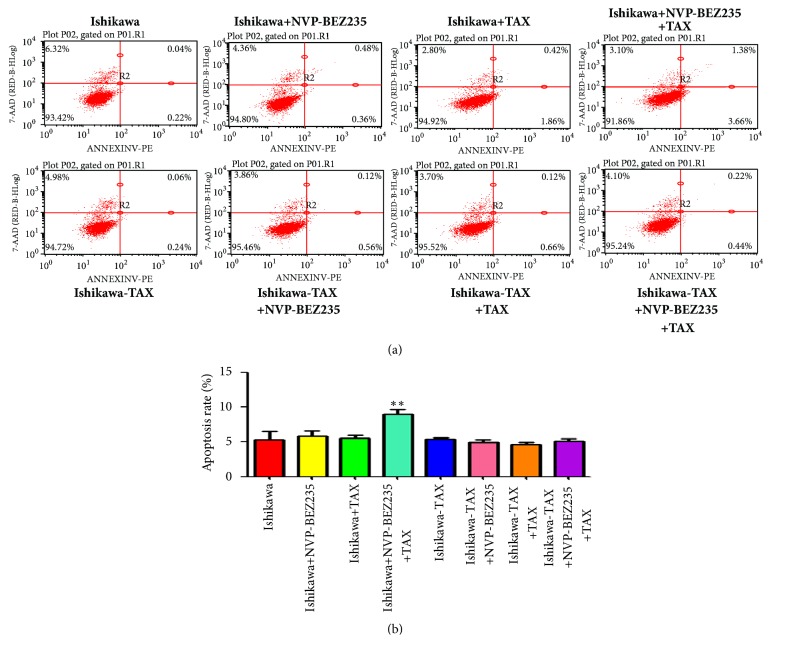
The apoptosis of Ishikawa and Ishikawa-TAX cells after treatment of NVP-BEZ235 and paclitaxel. (a) NVP-BEZ235 alone and cotreated with paclitaxel Ishikawa and Ishikawa-TAX cells and the changes in apoptosis. (b) Apoptosis quantification (*∗∗*P <0.01).

Apoptosis results showed that cotreatment of NVP-BEZ235 with paclitaxel significantly increased apoptosis of Ishikawa, while cotreatment of NVP-BEZ235 with paclitaxel did not result in significant apoptosis of Ishikawa-TAX (Figure 4)

### 3.5. NVP-BEZ235 Cotreatment with Paclitaxel Increased Ishikawa and Ishikawa-TAX Cells in the Proportion of G1 Phase

**Figure 5:**
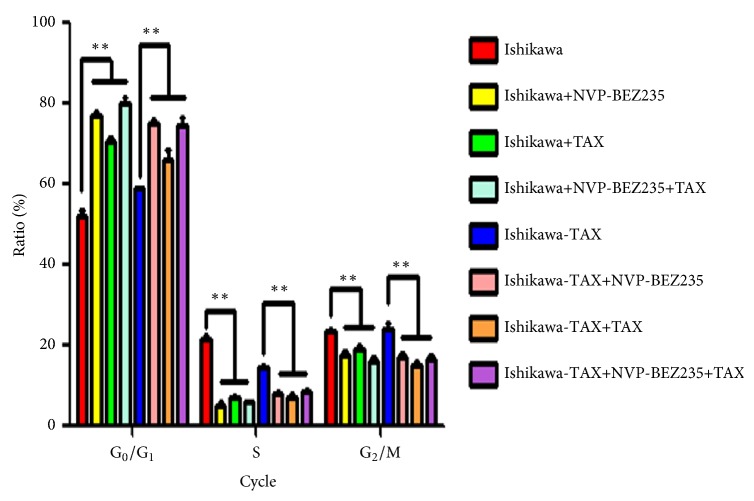
Cell cycle changes after NVP-BEZ235 and paclitaxel treatment of Ishikawa and Ishikawa-TAX cells. The cell cycle of Ishikawa and Ishikawa-TAX cells changes of G0/G1, S, G2/M cycles after NVP-BEZ235 and paclitaxel alone and in cotreatment (*∗∗*P <0.01).

The results of cell cycle showed that NVP-BEZ235 and paclitaxel alone increased the proportion of Ishikawa and Ishikawa-TAX cells in G1 phase, while NVP-BEZ235 cotreatment with paclitaxel further increased the proportion of Ishikawa and Ishikawa-TAX cells in G1 phase (Figure 5)

### 3.6. NVP-BEZ235 Cotreatment with Paclitaxel Increased Ishikawa and Ishikawa-TAX Cells p-AKT and p-p70 S6 K Protein Expression

**Figure 6:**
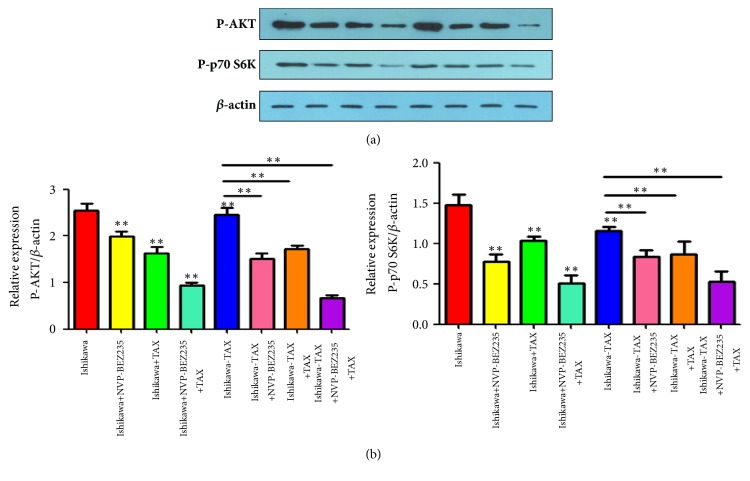
Changes of p-AKT and p-p70 S6K proteins after treatment with NVP-BEZ235 and paclitaxel in Ishikawa and Ishikawa-TAX cells. (a) Changes of p-AKT and p-p70 S6K proteins after treatment of Ishikawa and Ishikawa-TAX cells with NVP-BEZ235 and paclitaxel alone and in combination. (b) Protein relative quantification (*∗∗*P <0.01).

Western blotting results showed that NVP-BEZ235 and paclitaxel alone treatment reduced p-AKT and p-p70 S6K protein levels in Ishikawa and Ishikawa-TAX cells, while NVP-BEZ235 cotreatment with paclitaxel could further reduced p-AKT and p-p70 S6K protein expression (Figure 6).

### 3.7. LY294002 Cotreatment with Paclitaxel Can Significantly Lead to the Increase in Apoptosis Rate and the Proportion of G1 Phase

**Figure 7:**
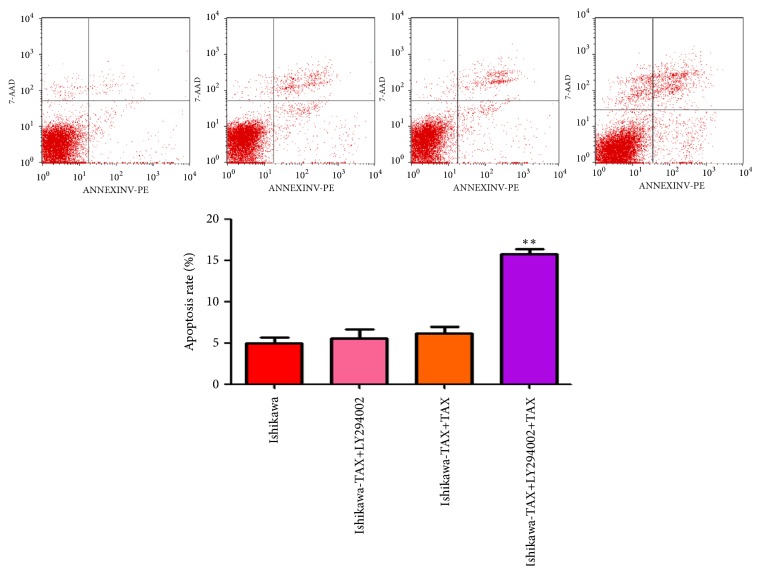
The apoptosis of Ishikawa-TAX cells after treatment of LY294002 and paclitaxel. (a) LY294002 alone and cotreated with paclitaxel Ishikawa-TAX cells, the changes in apoptosis. (b) Apoptosis quantification (*∗∗*P <0.01).

**Figure 8:**
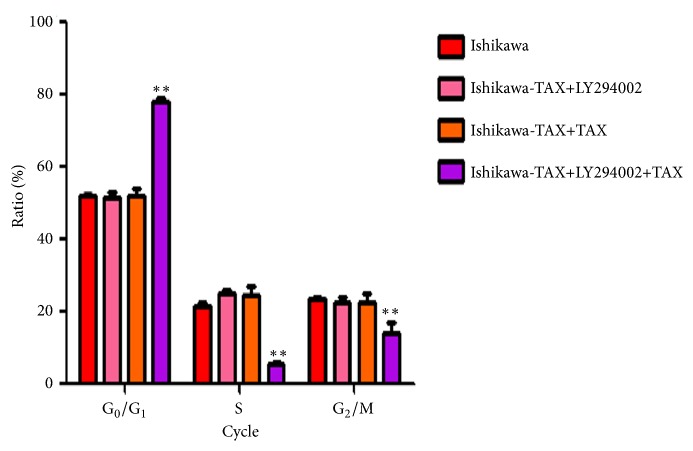
Cell cycle changes after LY294002 and paclitaxel treatment of Ishikawa-TAX cells. The cell cycle of Ishikawa-TAX cells changes of G0 / G1, S, G2 / M cycles after LY294002 and paclitaxel alone and in cotreatment. (*∗∗*P <0.01).

Apoptosis results showed that cotreatment of LY294002 with paclitaxel significantly increased apoptosis of Ishikawa-TAX, while the treatment of LY294002 or paclitaxel did not result in significant apoptosis of Ishikawa-TAX (Figure 7). The results of cell cycle showed that cotreatment of LY294002 and paclitaxel increased the proportion of Ishikawa-TAX cells in G1 phase (Figure 8).

### 3.8. LY294002 Cotreatment with Paclitaxel Decreased the Expression of p-AKT and p-p70 S6 K Protein in Ishikawa-TAX Cells

**Figure 9:**
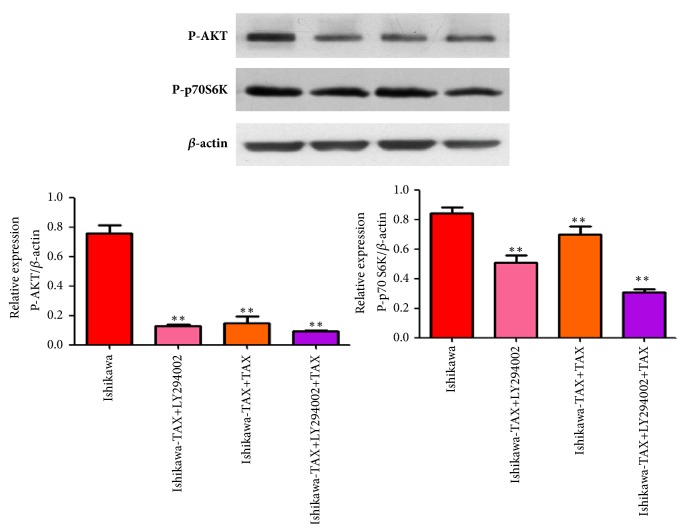
Changes of p-AKT and p-p70 S6K proteins after treatment with LY294002 and paclitaxel in Ishikawa-TAX cells. (a) Changes of p-AKT and p-p70 S6K proteins after treatment of Ishikawa-TAX cells with LY294002 and paclitaxel alone and in combination. (b) Protein relative quantification (*∗∗*P <0.01).

Western blotting results showed that LY294002 and paclitaxel alone treatment reduced p-AKT and p-p70 S6K protein levels in Ishikawa-TAX cells, while LY294002 cotreatment with paclitaxel could further reduced p-AKT and p-p70 S6K protein expression (Figure 9).

## 4. Discussion

Endometrial cancer is a malignant tumor which originates from endometrial glands with a high incidence. It accounts for 20%-30% of the female reproductive system malignant tumors, which seriously endangers women's health and life [14, 15]. Chemotherapy is the common treatment for endometrial cancer and the sensitivity of tumor cells makes a big difference in the prognosis. Improving sensitivity and reducing of drug resistance will raise the survival rate of endometrial cancer and therefore improve the patients' life quality [16, 17]. Paclitaxel is one of the most commonly used chemotherapy drug, which can be applied in endometrial cancer, colon cancer, and other malignancies [18–20]. However, in clinic, emergence of paclitaxel resistance greatly affects its effect and restricts its application as a chemotherapeutic drug.

In this study, we used the taxol concentration gradient method to establish the paclitaxel resistant cell line (Ishikawa-TAX cells) by repeated induction of Ishikawa parent cells. Through its IC50, resistance-related genes (Pgp and MDR1) were detected, which confirmed that the constructed cell line was Ishikawa-TAX (Figure 1). The results showed that Pgp and MDR1 were increased in Ishikawa-TAX cells, which indicated that it was a promising therapy to focus on the genes of Pgp and MDR1 in Ishikawa-TAX cells. So far, the mechanism of Ishikawa cells' resistance to paclitaxel has not been clearly illustrated and need further study.

The phosphatidylinositol-3-kinase (PI3K)/Akt/mTOR pathway is a commonly activated signaling pathway in cancer, which play an important role in drug resistance of cancers, including prostate cancer and lung cancer [21, 22], while the relationships between Ishikawa and taxol is still unclear. In this study, we used NVP-BEZ235, which is the inhibitor of PI3K/Akt/mTOR pathway [23]. NVP-BEZ235 is also a promising chemotherapy drug in clinical trials for many cancers such as colon cancer [24, 25].

Compared with paclitaxel alone, the combined treatment of NVP-BEZ235 and paclitaxel further enhanced the inhibitory effect on the clone formation and invasion of Ishikawa and Ishikawa-TAX cells (Figures 2 and 3). This suggests that the inhibitory effect of taxol on Ishikawa and Ishikawa-TAX is mediated by the PI3K/mTOR signaling pathway. In apoptosis detection, the combined treatment of NVP-BEZ235 and paclitaxel showed similar result and raised the apoptosis induction effect of taxol on Ishikawa cells (Figure 4). The combined treatment also enhanced the arrest of G0/G1 cells in Ishikawa and Ishikawa-TAX cells. These results collectively showed that the cotreatment of NVP-BEZ235 and paclitaxel could lead to a decrease in the proliferation activity of Ishikawa and Ishikawa-TAX cells (Figure 5). When we looked into the underlying mechanism, we found that simple paclitaxel treatment could significantly inhibit the expression of p-AK and p-p70 S6K protein in Ishikawa and Ishikawa-TAX cells. When combined with paclitaxel, NVP-BEZ235 can further inhibit the expression of p-AKT and p-p70 S6K protein in Ishikawa and Ishikawa-TAX cells, indicating that paclitaxel inhibits Ishikawa and Ishikawa-TAX cells through PI3K/Akt/mTOR (Figure 6). LY294002, an inhibitor of PI3K-AKT pathway, was used to further confirm the function of AKT pathway. Results showed that the apoptosis rate and cell cycle were not different between Ishikawa group and Ishikawa-TAX + TAX group, which suggested that Ishikawa-TAX had the ability of resistance for paclitaxel (Figures 7 and 8). What is more, the results showed that paclitaxel inhibits Ishikawa-TAX cells through PI3K/Akt/mTOR (Figures 7, 8, and 9).

Nowadays, more and more clinical trials focus on the combination of paxlitaxel with other PI3K inhibitors in cancer treatment, such as rapamycin and TORC1/2 inhibitor TAK-228 [26, 27]. It shows that PI3K pathway plays an important role in cancer growth, invasion, and drug resistance.

In summary, taxol treatment can inhibit the Ishikawa cells and Ishikawa-TAX cells through PI3K/Akt/mTOR signaling pathway, and therefore regulating PI3K/Akt/mTOR pathway may been a promising therapeutic direction for the clinical treatment of drug-resistant endometrial carcinoma.

## Data Availability

The data used to support the findings of this study are available from the corresponding author upon request.
